# The Sentiworld project: global mapping of sentinel surveillance networks in general practice

**DOI:** 10.1186/s12875-022-01776-x

**Published:** 2022-07-14

**Authors:** Andrew Meci, Florence Du Breuil, Ana Vilcu, Thibaud Pitel, Caroline Guerrisi, Quentin Robard, Clément Turbelin, Thomas Hanslik, Louise Rossignol, Cécile Souty, Thierry Blanchon

**Affiliations:** 1 INSERM, Institut Pierre Louis d’Epidémiologie et de Santé Publique, IPLESP, UMRS 1136, Sorbonne Université, F75012 Paris, France; 2 Université de Versailles Saint-Quentin-en-Yvelines, UVSQ, UFR Simone Veil - Santé, F78180 Montigny-le-Bretonneux, France; 3 Assistance Publique - Hôpitaux de Paris, APHP, Hôpital Ambroise Paré, Service de Médecine Interne, F92100 Boulogne-Billancourt, France; 4 Université de Paris, Faculté de Médecine, Département de médecine générale, Université Paris Diderot, F75018 Paris, France

**Keywords:** General practice, Family practice, Sentinel surveillance, Epidemiology, Health status indicators, Global health

## Abstract

**Background:**

Sentinel networks composed of general practitioners (GPs) represent a powerful tool for epidemiologic surveillance and ad-hoc studies. Globalization necesitates greater international cooperation among sentinel networks. The aim of this study was to inventory GP sentinel networks involved in epidemiological surveillance on a global scale.

**Methods:**

GP sentinel surveillance networks were inventoried globally between July 2016 and December 2019. Each identified network was required to fill out an electronic descriptive survey for inclusion.

**Results:**

A total of 148 networks were identified as potential surveillance networks in general practice and were contacted. Among them, 48 were included in the study. Geographically, 33 networks (68.8%) were located in Europe and 38 (79.2%) had national coverage. The number of GPs registered in these networks represented between 0.1 and 100% of the total number of GPs in the network’s country or region, with a median of 2.5%. All networks were involved in continuous epidemiologic surveillance and 47 (97.9%) monitored influenza-like illness. Data collection methods were paper-based forms (*n* = 26, 55.3%), electronic forms on a dedicated website (*n* = 18, 38.3%), electronic forms on a dedicated software program (*n* = 14, 29.8%), and direct extraction from electronic medical records (*n* = 14, 29.8%). Along with this study, a website has been created to share all data collected.

**Conclusions:**

This study represents the first global geographic mapping of GP sentinel surveillance networks. By sharing this information, collaboration between networks will be easier, which can strengthen the quality of international epidemiologic surveillance. In the face of crises like that of COVID-19, this is more imperative than ever before.

## Introduction

Epidemiologic disease surveillance, defined by the World Health Organisation (WHO) as “the systematic collection and use of epidemiologic information for the planning, implementation, and assessment of disease control” [1, 2], consists of data collection over a specific period of time and providing prompt feedback to healthcare administrators in order to adjust health policies accordingly.

In many public health systems, the major pillars of epidemiologic surveillance are mandatory disease reporting and sentinel surveillance networks [3]. Supplemental data are also collected from more modern tools that are used in tandem. These tools include administrative databases [3], drug sales data [4], and participatory syndromic surveillance [5].

Sentinel epidemiologic surveillance relies on dedicated healthcare investigators, often in general practice, who report real-time data on specific health indicators. The objective of this type of surveillance is the early detection of unusual patterns of disease activity, estimation of the burden of disease in the population, and comparison of disease trends with historical data using data that is consistently collected by dedicated reporters using set case definitions [1, 3, 6].

Differences between sentinel networks include the health indicators monitored, representation of populations covered, types of reporters, methodologies for data collection and analysis, case definitions, and frequencies of reporting. Although general guidelines and case definitions are issued by international public health organizations, such as the European Centre for Disease Control and Prevention (ECDC) [7] and the WHO [8, 9], local epidemiological and public health surveillance systems often adapt these guidelines according to their health care system’s specificities and public health objectives. Further, while the International Health Regulations 2005 is a legally binding agreement to combat global epidemics, they pertain mostly to mandatory disease reporting systems, and not to sentinel networks [10, 11]. Sentinel data comparison across geographic areas and data pooling requires adequate knowledge of the available sentinel networks as well as knowledge of the methodology by which data are collected. Understanding this heterogeneity is crucial for improved disease surveillance at higher geographical levels [12].

Several past projects have attempted to identify and describe the sentinel networks that exist at the European level. The first inventories of sentinel networks were conducted in 1987 and 1990 and resulted in a group of 15 European sentinel research networks of general practitioners (GP) called Eurosentinel [13]. Further studies in 2003 and 2006 sought to update this inventory on the European scale and further document the surveillance activities of these networks [14, 15]. Other studies have been carried out by European organizations and institutions, including an inventory carried out by the Euvac project in 2001 [16, 17] and by the ECDC and WHO [18, 19]. However, these surveyed only European networks, focusing mostly on influenza, and are all more than a decade old (with the exception of an up-to-date list in the FluNews project, but this data is restricted to only previously identified European networks [19]). Additionally, epidemiologic surveillance based on primary health care providers acting as sentinel investigators has significantly evolved over the past decades, particularly due to the advent of electronic medical records (EMR) and the advancements in automatic data collection based upon electronic record frameworks.

Inter-network cooperation has long been described as essential at the European level [14], especially for comprehensive flu surveillance [20]. An updated inventory on a global scale would expand upon past works and take a step toward this necessity. For this purpose, the Sentiworld project was launched in 2016 with the objective of creating an inventory of sentinel surveillance networks in general practice at a global scale and providing a public online platform that shares collected network data. Since this time, with the emergence of new global public health crises, such as the COVID-19 pandemic, the need for this cooperation is even more apparent. We envision this project to be a resource that allows for a greater understanding of the structures and operations of different sentinel networks, while facilitating interaction and scientific collaboration between networks, thus taking a step toward improving global disease surveillance. In this study, we present the Sentiworld project and the characteristics of the participating networks.

## Methods

A descriptive study of sentinel surveillance and health research networks was first conducted in Europe and was then extended progressively to a global scale. This study was conducted using the resources of the French national sentinel surveillance network [21]. The study period was July 2016 to December 2019.

For inclusion in this study, networks had to be: (1) Sentinel-based surveillance networks, (2) composed, at least partially, of general practitioners, (3) monitoring a whole country or specific region of a country, (4) collecting health data on an ongoing or regular basis, and (5) having a goal of health surveillance or research.

The first step consisted of identifying potential surveillance networks in general practice. For this task, we relied initially on previous work on this subject [13–15, 17, 18]. We then searched for research articles and surveillance reports from different countries on a global scale in order to identify other surveillance networks [22–25]. We also contacted epidemiological surveillance experts in Europe and worldwide for assistance in identifying sentinel surveillance networks. These contacts included partners of the I-Move (Influenza-Monitoring of Vaccine Effectiveness) [26] and InfluenzaNet projects [27], as well as ECDC members. Finally, internet and literature research was conducted to identify sentinel surveillance networks, especially outside of Europe.

### Online questionnaire

Identified potential GP sentinel networks were invited to fill an electronic survey in English. The questionnaire was generated on the LimeSurvey platform and distributed by email. This survey gathered descriptive information from each network in categories such as: organization (date of creation, name, support structure, main contact information), investigators (medical specialty, number, tasks carried out for the network, compensation, representation), epidemiological surveillance (monitored indicators, frequency and collection method, frequency and method for transmission data), data availability, and data accessibility for use in collaboration with other organizations nationally or internationally.

In cases of data gaps, we reached out directly to networks for additional information or clarification. All direct contact with networks was conducted via email or over the phone. For some networks, partial information was completed after further research was conducted on the web or in scientific literature.

Responses to questionnaires (officially submitted to our system) were reviewed for completeness and satisfaction of inclusion requirements. Networks not meeting inclusion requirements were excluded from the study.

### Data analysis

Using the collected data, we performed a descriptive analysis. We described quantitative data by their mean, median, minimum, and maximum. Quantitative data were date of network creation, number of general practitioners (GPs), and percentage of the country’s total GPs represented by those participating in the network. We described non-numeric data with the number and percentage for each response category recorded. Non-numeric data were geographical coverage, continent, support structure for the network, network funding, investigator specialties, financial compensation, representation of network investigators proportionate to the total number of practitioners in the coverage region, and tasks of network investigators. Analyses were carried out using R version 3.5.1 [28].

### Public online platform

Following data collection and analysis, we developed a website called Sentiworld to share collected data with epidemiological surveillance stakeholders worldwide and with the public. The web platform was developed using the PSP Symfony framework and the data are stored in a SQL database. The site has two portions with differing requirements for access: an administrative section accessible with a network key for page creation and management, and a public section for access to available network information.

## Results

### Participation

**Fig. 1:**
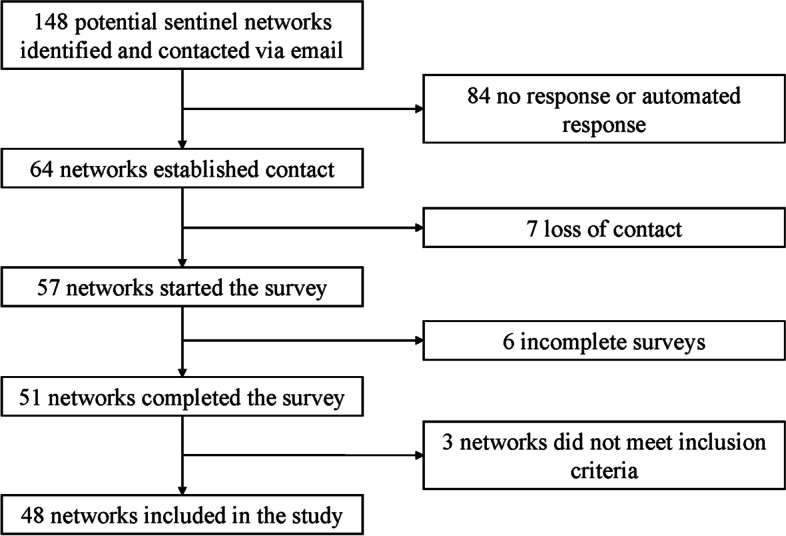
Flowchart of networks identified and included in the Sentiworld study

In total, 148 potential networks in 116 countries were identified and contacted electronically. Contact was established with 64 networks (43.2%). Response rates by continent were 36.0% for Africa (9/25), 6.9% for Asia (2/29), 68.4% for Europe (39/57), 53.9% for North America (7/13), 33.3% for Oceania (3/9), and 26.7% for South America (4/15), in comparing contacts established with the total number of networks contacted in that continent. Among networks with which contact was established, 51 networks (79.7%) in 35 different countries replied to the questionnaire and were considered for this study, with 48 meeting the inclusion criteria (Fig. 1).

### Network descriptions

**Fig. 2:**
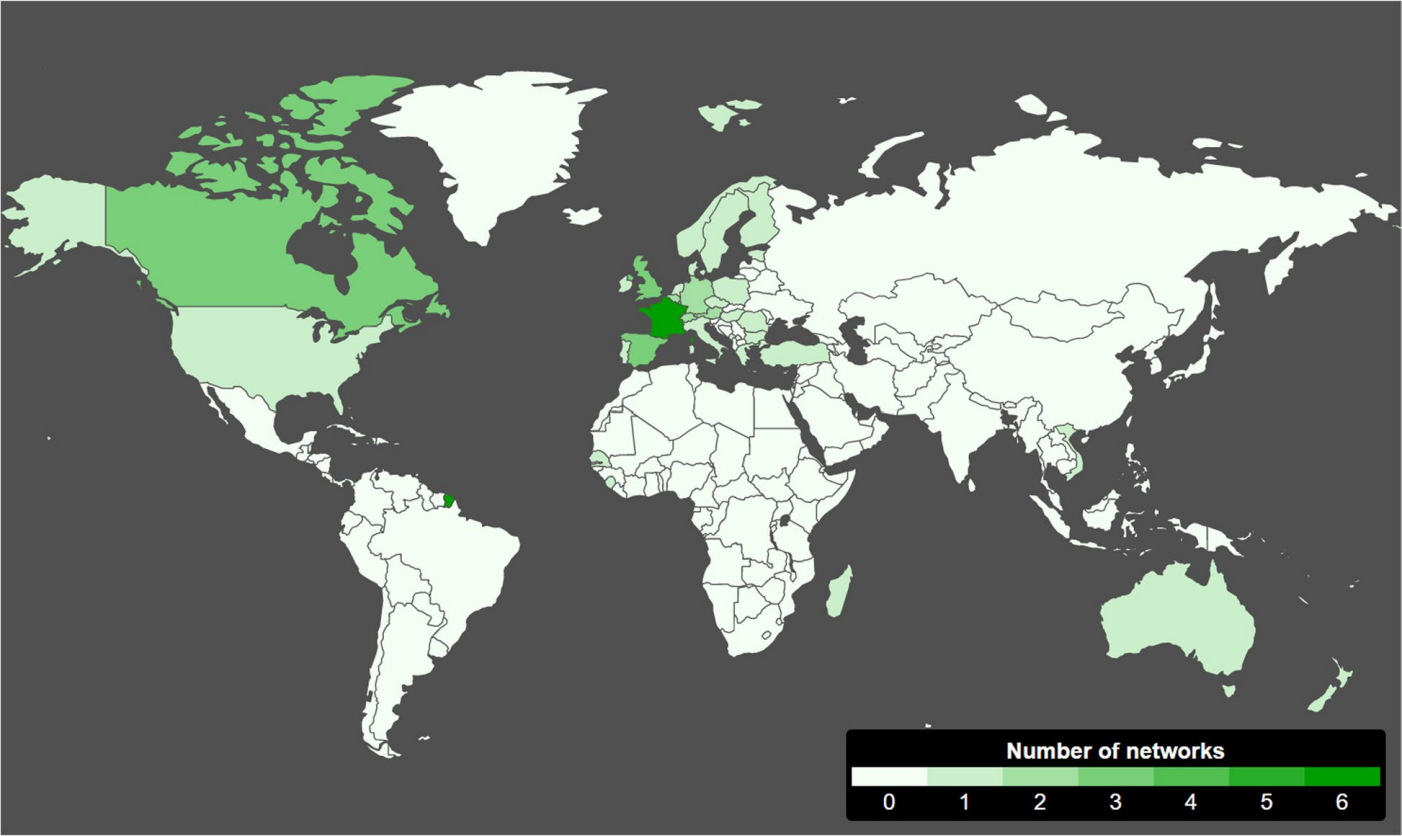
Global mapping of GP sentinel surveillance networks included in the Sentiworld study. This image was taken from our Sentiworld website (©*Réseau Sentinelles*), developed with PSP Symfony

Geographically, 33 networks (68.8%) were located in Europe (Table 1, Fig. 2). The other continents represented were North America (*n* = 5, 10.4%), Africa (n = 5, 10.4%), Oceania (*n* = 3, 6.2%), Asia (*n* = 1, 2.1%), and South America (n = 1, 2.1%). Among the networks included in this study, 38 (79.2%) had national surveillance coverage, 8 (16.7%) had regional coverage, and 2 (4.2%) covered municipalities.

**Table 1:** General description of 48 sentinel surveillance networks included in the Sentiworld study

	n	%
Continent (*m.d.*^a^ *= 0)*		
Europe	33	68.8
North America	5	10.4
Africa	5	10.4
Oceania	3	6.2
Asia	1	2.1
South America	1	2.1
Geographic Coverage (*m.d. = 0)*		
National	38	79.2
Regional	8	16.7
Municipality	2	4.2
Structure on which the network depends *(m.d. = 0) (multiple structures per network are possible)*		
Government structure	36	75.0
Public university structure	8	16.7
Public research structure	7	14.6
Other structures ^b^	4	8.3
Finances (*m.d. = 0)*		
Public funds only	43	89.6
Private funds only	1	2.1
Mixed funds	4	8.3

^a^*m.d.*= missing data

^b^include “independent research institute, not-for-profit foundation”, “municipality”, and “private research structure” (*n* = 2)

**Fig. 3:**
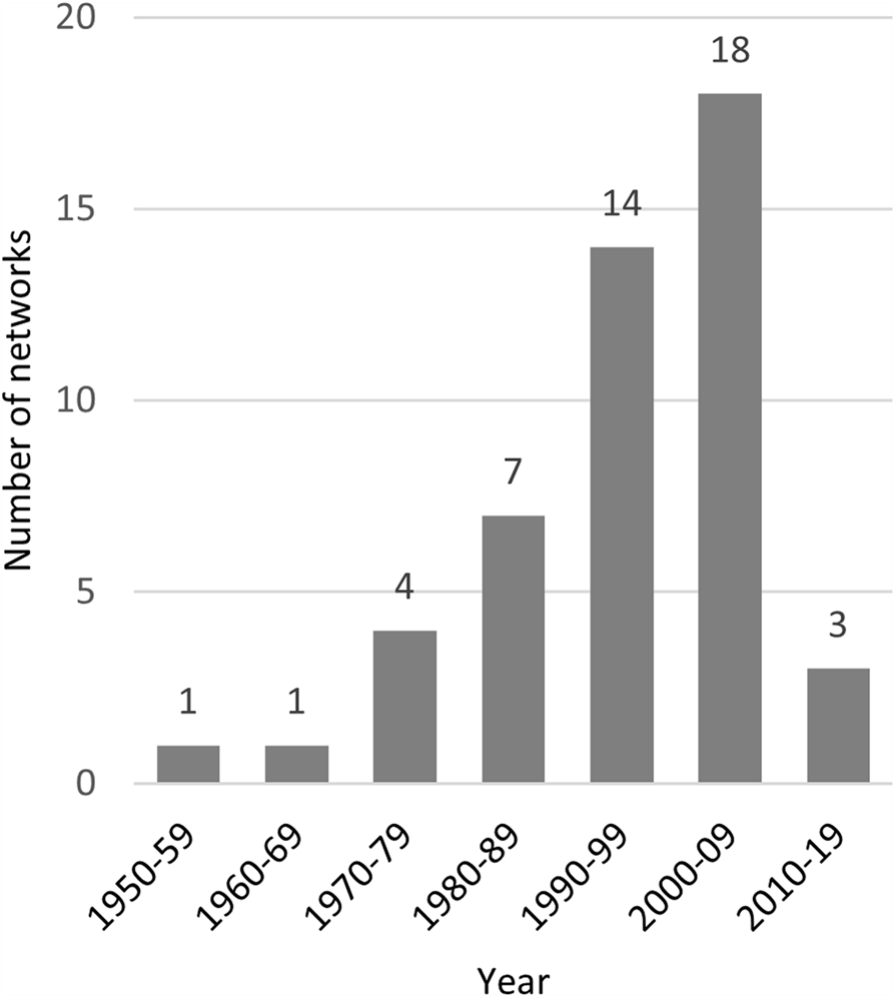
Year of creation for networks grouped in age bands of 10 years

The earliest year of creation was 1951 while the latest was 2017. Almost half of all included networks were created in the year 2000 or later (*n* = 22, 45.8%), and 18 (37.5%) were created between 2000 and 2009 (Fig. 3).

Regarding structural organization and financial support, the majority of the networks were relied on government structures (like a national health department) (*n* = 36, 75.0%) and a vast majority relied exclusively on public funding (*n* = 43, 89.6%) (Table 1).

### Investigator descriptions

The median number of general practitioners per network was 140, ranging from 3 (Sierra Leone) to 2600 (United States) (Table 2). The proportion of general practitioners registered in the network to the total number of general physicians in the network’s country or region was between 0.1 and 100%, with a median of 2.5%. Twenty-five networks (62.5%) had studied the representativeness of their sentinel investigators. Many networks also included investigators from specialties other than general practice, such as paediatricians (*n* = 13, 27.1%) and nurses (*n* = 10, 20.8%).

**Table 2:** Investigator descriptions for the 48 networks included in the Sentiworld study

	n	%
Number of investigators by type *(m.d.*^a^ *= 0)*		
General practitioners	48	100
Paediatricians	13	27.1
Nurses	10	20.8
Hospital Physicians	7	14.6
Microbiologists	5	10.4
Internists	3	6.2
Number of GPs registered per network *(m.d. = 4)*		
Minimum	3	
Maximum	2600	
Mean	403	
Median	140	
Number of GPs participating in the current year *(m.d. = 4)*		
Minimum	3	
Maximum	2600	
Mean	368	
Median	130	
Percentage of country’s GPs registered in the network *(m.d. = 7)*		
Minimum		0.1
Maximum		100
Mean		12.7
Median		2.5
Study of GPs representation in the network *(m.d. = 8)*		
Yes	25	62.5
Investigator activities *(m.d. = 0)*		
Continuous epidemiologic surveillance	48	100
Ad-hoc epidemiologic studies	10	20.8
Clinical Trials	2	4.2
Financial compensation for GPs *(m.d. = 0)*		
Yes	14	29.2
No	34	70.8
Compensation according to the activity *(m.d. = 0)*		
Epidemiologic surveillance (*n* = 48)	13	27.1
Ad-hoc epidemiologic studies (*n* = 10)	1	10.0
Clinical Trials (*n* = 2)	1	50.0

^a^*m.d.* missing data

The principal task of investigators was to carry out continuous epidemiologic surveillance of certain health indicators (*n* = 48, 100%). Networks also engaged in ad-hoc epidemiological studies (*n* = 10, 20.8%) and clinical trial studies (*n* = 2, 4.2%). Almost a third of networks provided compensation for their investigators (*n* = 14, 29.2%) (Table 2).

### Description of Health Indicators

Among the 48 networks that carried out epidemiological surveillance, all 48 (100%) studied infectious disease indicators, and 5 (10.4%) studied non-infectious disease indicators. Eleven (22.9%) investigated other health indicators (Table 3).

**Table 3:** Organisation of the sentinel surveillance in the 48 networks included in the Sentiworld study

	n	%
Heath indicators followed (*m.d.*^a^ *= 0)*		
Infectious disease indicators	48	100
Influenza-like-infections (ILI)	47	97.9
Acute respiratory infections (ARI)	19	39.6
Gastroenteritis	15	31.2
Diarrhoea	12	25.0
Varicella	12	25.0
Mumps	8	16.7
Pertussis	8	16.7
Shingles	6	12.5
Lyme disease	6	12.5
Dengue	6	12.5
Measles	6	12.5
Rubella	6	12.5
Urethritis	5	10.4
Malaria	4	8.3
Other infectious disease indicators	19	39.6
Non-infectious disease indicators	5	10.4
Other health indicators^b^	11	22.9
Mode of data collection *(m.d. = 1) (multiple modes per network are possible)*		
Paper-based form	26	55.3
Electronic form on a dedicated website	18	38.3
Electronic form on a dedicated software	14	29.8
Extraction from Electronic Medical Records	14	29.8
Mode of data transmission *(m.d. = 1) (multiple modes per network are possible)*		
Internet (excluding e-mail)	32	68.1
Postal mail	25	53.2
Email	10	21.3
Phone (call or SMS)	8	17.0
Fax	5	10.6
Frequency of transmission *(m.d. = 0) (multiple frequencies per network are possible)*		
Daily	9	18.8
Weekly	35	72.9
Other^c^	7	4.4
Publication of an epidemiological report *(m.d.*^a^ *= 0)*		
Yes	47	97.9
If yes, established frequency *(m.d.*^a^ *= 7) (multiple frequencies per network are possible)*		
Daily	2	5.0
Weekly	33	82.5
Monthly	2	5.0
Annually	3	7.5
Other^d^	5	12.5
If yes, medium of publication *(m.d. = 6) (multiple medium per network are possible)*		
Website	38	92.7
Email	2	4.9
Paper	3	7.3
Availability of data for outside research *(m.d. = 3)*		
Yes	33	73.3

^a^*m.d.* missing data

^b^ include “suicide or suicide attempts” (*n* = 6, 12.5%)

^c^ Frequencies: Dependent on health indicators (3), every 3 months (1), yearly (1), when they want without exceeding a surveillance period of 12 days (1), and unspecified (1)

^d^ Other time intervals were quarterly (2), variable based on province, every 2 weeks, and every 3 years

Regarding infectious disease indicators, 47 (97.9%) continually surveyed influenza-like illness and 19 (39.6%) surveyed acute respiratory infections. Other infectious health indicators frequently surveyed were gastroenteritis (*n* = 15, 31.2%), acute diarrhoea (*n* = 12, 25.0%) and varicella (n = 12, 25.0%) (Table 3). Non-infectious disease indicators surveyed include chronic diseases such as asthma (*n* = 3, 6.3%) and diabetes (n = 3, 6.3%). Other health indicators surveyed include suicide or suicide attempts (*n* = 6, 12.5%).

Twenty-six networks (55.3%) collected data using paper-based forms, 18 (38.3%) used an electronic form on a dedicated website, 14 (30.4) used an electronic form on a dedicated software program, and 14 (29.8%) extracted data directly from electronic medical records (Table 3). Further, 46 networks (95.8%) had established frequencies for data transmission.

### Publication of epidemiologic surveillance data

All 48 networks published their data regularly (Table 3). Additionally, 33 networks (73.3%) allowed for their collected data to be used by outside researchers and organizations, often having specific procedures for data requests.

## Discussion

This work presents the characteristics of 48 general practitioner sentinel surveillance networks from around the world. To the best of our knowledge, this study is the first to take inventory of sentinel surveillance networks in general practice on a global scale.

Building on past inventories on the European scale, the Sentiworld inventory of networks has found novel networks in addition to those previously recorded [13–19]. Many of these previously studied sentinel networks have establishment dates in the mid to late twentieth century, suggesting that these are durable structures that have been able to adapt to changing public health landscapes (Fig. 2). Furthermore, 22 networks were created in 2000 or later, including networks from countries that were not included in previous works. The creation of new sentinel networks both in nations where other such networks did and did not previously exist and the emergence of networks that are monitoring a more varied array of health indicators indicates meaningful, progressive change in the field of epidemiological surveillance.

Previous work by Deckers et al. in 2006 set a threshold of > 1% for the inclusion of general practitioners in sentinel networks [14]. Over two-thirds of included networks had a percentage of general practitioners participating in the network greater than or equal to 1%. Some networks went further and have profiled their sentinel practitioners in terms of regional and demographic representation. This has been noted to help improve network functionality, as gaps in coverage of the population are better and more specifically known [29].

The main and often exclusively monitored health indicator is influenza. Previous studies support this finding [14, 15, 18, 19]. However, our study also shows that networks monitor many other health indicators, including infectious diseases that cause acute health problems (outside of influenza), non-infectious (chronic) disease incidences, and other, non-disease indicators such as suicides.

Data collection for sentinel surveillance purposes has evolved over time. Networks have trended from paper forms to electronic resources like dedicated websites and software. In recent years, with the wider use of EMR, sentinel epidemiology has trended towards the use of direct, automatic data extraction from these records to be used in tandem or in place of traditional sentinel collection mediums [30, 31]. In theory, EMR systems make many improvements over traditional reporting methods, as they provide data on more patients in a longitudinal sense and on more indicators [30]. Access to EMR data is also more rapid and has been shown to reduce the risk errors in disease surveillance [32]. We have found from our own data that EMR data collection does in fact have a strong positive correlation with the number of indicators surveyed, the surveillance of non-infectious indicators, and non-infectious indicator surveillance. This is likely because of the larger scope of data contained within EMR. EMR surveillance data is also better standardized than traditional sentinel data and thus could be a solution to homologizing sentinel surveillance, given the diversity of networks included in the Sentiworld study. However, EMR data collection cannot be a fix-all solution to all gaps in sentinel surveillance nor in international collaboration, as EMR data collection is also complicated in places where many private companies control medical data with data systems, coding or diagnoses are unstandardized, or data that is unsuited for epidemiological modelling.

Our results show that sentinel networks, despite all existing in general practice, appear to be heterogeneous. It is thus crucial to understand the differences between nations in healthcare management, in the laws and practice of general medicine, and in population health habits [33] when engaging in global health collaborations. It is also necessary to work towards a better understanding of the differences between networks, including case definitions, when comparing and sharing data. As of this moment, limited collaboration between sentinel networks is taking place, even in Europe where sentinel networks are most plentiful and established, and even in the face of the COVID-19 pandemic. This can be attributed to a lack of data homogeneity (compared to mandatory reporting systems) as well as an even broader lack of comprehension of foreign sentinel surveillance and health systems. Despite the challenges posed by international collaboration, it is important that this cooperation exists in our globalising society in where it is possible to fly from one part of the world to another in less time than the incubation period for most infectious diseases [10]. This idea held true certainly at the start of the Sentiworld project in 2016 and does so even more in 2021, amidst the worst public health crisis in a century, COVID-19. In this time, we have seen unprecedented collaboration among healthcare professionals, researchers, and surveillance systems across borders and continents [34]. With sentinel systems necessary to supplement research and mandatory surveillance data in the context of infection management, policy decisions, and vaccine monitoring, we are again confronted with the barrier of heterogeneity between systems. Our cartography allows networks to learn more about each other, which bridges many gaps in information that previously existed and allows for potentially greater inter-network cooperation.

One of the major strengths of this study is that it is the first of its kind in many respects. First, it extends its area of study to a global scale by contacting 148 networks in 116 countries over 6 continents. Despite a low overall response rate of 43.2%, compared to other studies of the like, we contacted many more networks, and were able to include many outside of Europe. Additionally, because data was supplied by network administrators, they were more likely to be reliable and exact. Data sharing on our website also improves network visibility and improves cooperation among networks.

However, there are also limitations present in our study. Despite contacting many networks outside of Europe, the surveillance networks included in this study were largely European. Because we are based in France and limited linguistically to French and English, it was difficult to gather data from countries that were geographically distant, had vastly different health systems, or were less involved in research projects and, thus, more difficult to reach. Difficulty in finding accurate, up-to-date network contact information online often hampered efforts to expand our study as well. Additionally, even when contact information was found, data on the networks may have been scant, leading to doubt that even with a higher response rate, all of our 148 potential sentinel networks contacted would have fit our inclusion criteria. Given the anticipated health system differences, we attempted to use a standardized electronic survey, but realize that it was likely not adapted for all types of sentinel networks, especially those that differed greatly from our own or had more limited knowledge of English. Overcoming these linguistic, cultural, and technological challenges could have increased the number of included networks and broadened our geographic inclusion. Further, we were not able to engage in additional research to profile all physicians in our studied networks, which would have been interesting to add to this study. While not the subject of this study, further investigation could take place regarding the sensitivity of inter-network collaboration to detect early epidemic warning signals or a quantification of the value of cross-border collaboration or even international standardization of sentinel surveillance.

The findings of Sentiworld show that there is great potential for future work in the area of international collaboration. The heterogeneity of sentinel surveillance networks leads to questions about which systems are most effective and future studies could be focused on statistical associations among characteristics of sentinel networks in order to determine which methods of surveillance are most effective, at least in theory. Further, Sentiworld data and its website can be used as a tool for communication among sentinel networks. Understanding differences between sentinel networks could allow for easier sharing of data in future studies and when dealing with public health crises. In fact, correspondence that took place for the Sentiworld study later led to the formation of a small working group of eight sentinel surveillance networks in seven European countries in 2020. The group was later able to collaborate on early COVID-19 research on initial risk perception and sentiments of preparedness among primary care physicians regarding the COVID-19 pandemic [35].

## Creation of the Sentiworld website

The Sentiworld website [36] allows the public to consult the information pertaining to each network detailed in this article. Each network has a dedicated webpage that can be found on a world map on the website’s homepage, on a list of the networks, or by searching keywords using a search engine. The site is expected to grow and develop with the expansion of this project and with the refinement of its features. It will be updated on a yearly basis.

## Data Availability

The datasets generated and analysed during the current study are available at the Sentiworld website, http://sentiworld.sentiweb.fr/. The map in Fig. 2 is taken from our Sentiworld website.

## Abbreviations

GP: General practitioner
ECDC: European Centre for Disease Prevention and Control
WHO: World Health Organization
EMR: Electronic Medical Record
